# Supplementary material for: Rapid 3D phenotyping of chick embryo liver development at HH22-HH41 embryonic stages using X-ray microcomputed tomography with PTA staining
Source: PLoS One. 2025 Oct 27;20(10):e0333995. doi: 10.1371/journal.pone.0333995 (PMC12558506; doi:10.1371/journal.pone.0333995)
Supplement: Attachment — Submitted filename: Resposne to Reviewers.docx [file pone.0333995.s001.docx]

We appreciate the Editor and Reviewers for the time devoted for study of our manuscript and valuable comments and recommendations that helped us to improve the quality of our work. All comments were considered and decided correspondingly. All revised parts are marked by color. Please find point-by-point responses below.

Reviewer #1: The manuscript is scientifically sound, methodologically rigorous, and clearly presented.
Minor revisions are needed to enhance clarity and reproductibility.The imaging methodology is of broad potential interest for developmental biology and non-invasive phenotyping, especially in model organisms.

**Response:** Thank you very much for the positive response on our study. Regarding clarity and reproductibility we carefully checked the Materials and methods section for essential information on equipment, software and chemicals, as well as statistical data processing.

Reviewer #2: Very well executed and designed radiological study of liver morphogenesis. Figures comparing histological slides and uCT scans are really impressive. I recommend the manuscript for publishing since it is novel in the field of embryology.

**Response:** Thank you very much for the positive response on our study. We value it!

Reviewer #3: In their paper, 'Rapid 3D phenotyping of chick embryo liver development at HH22-HH41 embryonic stages using X-ray microcomputed tomography with PTA staining', the authors discuss the use of µCT analysis as a possible tool for monitoring changes in liver tissue during the development of the chick embryo as an animal model. The authors employed 1% phosphotungstic acid (PTA) as a contrast agent. This method has been used in other studies, but on different organs or animal models, or with a different concentration or duration of staining. The staining method was described in sufficient detail to allow it to be repeated. µCT images were visually compared directly with stained histological sections of embryos at corresponding developmental stages. By analysing the µCT images, the authors obtained data on liver and vascular volume at different stages of development. These data should have been summarised in Table 1, which the authors mention in the text but do not attach. The work is well written and the cited sources are up to date and relevant to the topic. The text is written in an intelligible fashion and in standard English.
Major issues:
Add Table 1, which contains data on liver and liver vessel volumes at various stages of chicken development.

**Response:** Thank you for your attentiveness and sorry for missing Table 1. Now it was attached

Minor issues:
1. I propose adding the units in which the liver volume and vessel volume values are expressed to the caption of Figure 1.

**Response:** Thank you for recommendation. Figure 1 was revised

2. I also propose replacing the term 'kidney' with 'metanephros' in the captions of the figures (Fig. 2, 6, 7, 8, 9, 10, 11, 12, 13 and 14), as used in Doaa et al. (2013) for similar developmental stages of chick embryos, for example. The term 'metanephros' more accurately describes the incomplete development of the kidney.
Doaa, M. Mokhtar; Enas, A. El-Hafez; Hassan, A. H.S; Fatma, A, Mostafa. Dynamics of Liver Development in Dandarawi Chicken. Journal of World's Poultry Research, Volume 3, Number 3, 2013, pp. 73-79(7)
**Response:** We accept the remark with gratitude. It is a good article, we referred to it in the materials and methods as a source on the basis of which we labeled and described the organs in 2.5. Histological preparation. It is clearly seen there that it is better to write metanephros, since the kidney at the stages of embryogenesis under consideration is still underdeveloped. We agree!

3. Finally, I suggest omitting the “quantitative” congruence with the histological and uCT analyses described in the 'Conclusions' section. The manuscript does not describe quantitative histological analysis, e.g. using stereology or image analysis of histological slides.

**Response:** Thank you for recommendation. We removed “qualitative” word
